# Chemokine-Like Receptor 1 Deficiency Does Not Affect the Development of Insulin Resistance and Nonalcoholic Fatty Liver Disease in Mice

**DOI:** 10.1371/journal.pone.0096345

**Published:** 2014-04-29

**Authors:** Nanda Gruben, Marcela Aparicio Vergara, Niels J. Kloosterhuis, Henk van der Molen, Stefan Stoelwinder, Sameh Youssef, Alain de Bruin, Dianne J. Delsing, Jan Albert Kuivenhoven, Bart van de Sluis, Marten H. Hofker, Debby P. Y. Koonen

**Affiliations:** 1 University of Groningen, University Medical Center Groningen, Department of Pediatrics, Molecular Genetics Section, Groningen, the Netherlands; 2 Utrecht University, Faculty of Veterinary Medicine, Dutch Molecular Pathology Center, Utrecht, the Netherlands; 3 Merck Research Laboratories, MSD, Department of Immune Therapeutics, Oss, the Netherlands; Institute of Medical Research A Lanari-IDIM, University of Buenos Aires-National Council of Scientific and Technological Research (CONICET), Argentina

## Abstract

The adipokine chemerin and its receptor, chemokine-like receptor 1 (*Cmklr1*), are associated with insulin resistance and nonalcoholic fatty liver disease (NAFLD), which covers a broad spectrum of liver diseases, ranging from simple steatosis to nonalcoholic steatohepatitis (NASH). It is possible that chemerin and/or *Cmklr1* exert their effects on these disorders through inflammation, but so far the data have been controversial. To gain further insight into this matter, we studied the effect of whole-body *Cmklr1* deficiency on insulin resistance and NAFLD. In view of the primary role of macrophages in hepatic inflammation, we also transplanted bone marrow from *Cmklr1* knock-out (*Cmklr1^-/-^*) mice and wild type (WT) mice into low-density lipoprotein receptor knock-out (*Ldlr^-/-^*) mice, a mouse model for NASH. All mice were fed a high fat, high cholesterol diet containing 21% fat from milk butter and 0.2% cholesterol for 12 weeks. Insulin resistance was assessed by an oral glucose tolerance test, an insulin tolerance test, and by measurement of plasma glucose and insulin levels. Liver pathology was determined by measuring hepatic inflammation, fibrosis, lipid accumulation and the NAFLD activity score (NAS). Whole-body *Cmklr1* deficiency did not affect body weight gain or food intake. In addition, we observed no differences between WT and *Cmklr1^-/-^* mice for hepatic inflammatory and fibrotic gene expression, immune cell infiltration, lipid accumulation or NAS. In line with this, we detected no differences in insulin resistance. In concordance with whole-body *Cmklr1* deficiency, the absence of *Cmklr1* in bone marrow-derived cells in *Ldlr^-/-^* mice did not affect their insulin resistance or liver pathology. Our results indicate that *Cmklr1* is not involved in the pathogenesis of insulin resistance or NAFLD. Thus, we recommend that the associations reported between *Cmklr1* and insulin resistance or NAFLD should be interpreted with caution.

## Introduction

Obesity is accompanied by the development of the metabolic syndrome, a cluster of metabolic abnormalities, which includes low-grade inflammation, dyslipidemia and insulin resistance. Since nonalcoholic fatty liver disease (NAFLD) is the hepatic manifestation of the metabolic syndrome, its increasing prevalence follows the increasing rates of obesity seen worldwide. As a result, NAFLD has become one of the main causes of chronic liver disease in Western societies [1]. NAFLD describes a broad spectrum of liver diseases, ranging from simple steatosis (intrahepatic fat accumulation) to nonalcoholic steatohepatitis (NASH), fibrosis, and cirrhosis [2]. NASH can be distinguished from simple steatosis by the presence of inflammation. It is unknown how NAFLD develops or which factors provoke its progression into NASH.

Recently, an adipokine named chemerin has been implicated in the metabolic syndrome and the progression of NAFLD. Chemerin was first identified as a chemo-attractant protein, attracting immune cells expressing the chemerin receptor chemokine-like receptor 1 (*Cmklr1*, also known as *ChemR23*) [3]. This suggests that chemerin has a pro-inflammatory role. However, studies investigating the role of *Cmklr1* in inflammation are controversial. *Cmklr1* knock-out (*Cmklr1^-/-^*) mice have been reported to be protected against central nervous system inflammation [4], but they were more susceptible to lipopolysaccharide-induced lung inflammation [5] and viral pneumonia [6]. As inflammation is thought to play a key role in the progression of insulin resistance and NAFLD, chemerin and *Cmklr1* may be involved in these disorders. Indeed, in several human populations, elevated plasma chemerin levels correlate positively with characteristics of the metabolic syndrome, including inflammation, insulin resistance, plasma lipids and body mass index [7]–[9]. In addition, elevations in serum chemerin levels were found in patients with NAFLD and NASH compared to healthy controls [10] and have been shown to positively correlate with markers of liver pathology, including fibrosis, portal inflammation and NAFLD activity score (NAS) [11]. In contrast, expression of chemerin in visceral adipose tissue in morbidly obese individuals correlated negatively with hepatic inflammation (unpublished data). Moreover, data on hepatic expression of *chemerin* and *Cmklr1* in human and mouse NAFLD are inconsistent, since both reduced and increased levels of these genes have been found [12]–[14]. Although these studies indicate that chemerin and its receptor may be involved in the pathogenesis of insulin resistance and NAFLD, a causative role remains to be established.

Thus far, only a few studies have investigated the effect of *Cmklr1* deficiency in metabolic disease and, similar to the studies investigating inflammation, the results have been controversial. In one study it was shown that *Cmklr1* deficiency induced glucose intolerance in mice [15], whereas another study showed no effect of *Cmklr1* deficiency on glucose intolerance [16]. In addition, *Cmklr1^-/-^* mice were found to have reduced hepatic steatosis on a low fat diet, but not on a high fat diet, whereas hepatic inflammation was reduced in *Cmklr1^-/-^* mice on both diets [15]. However, differences in inflammation were not confirmed by histological analyses and differences in body weight gain could not be excluded as a confounding factor in this study [15]. Moreover, despite the controversial role of *Cmklr1* deficiency in inflammation, the role of *Cmklr1* in NASH has not been investigated. In the current study, we investigated the effect of whole-body *Cmklr1* deficiency on insulin resistance and NAFLD. In view of the primary role of macrophages in NASH, we also transplanted bone marrow from *Cmklr1^-/-^* mice and wild type (WT) mice into low-density lipoprotein receptor knock-out (*Ldlr^-/-^*) mice. These mice develop hepatic inflammation when fed a high fat, high cholesterol (HFC) diet and can be regarded as a mouse model for NASH.

## Materials and Methods

### Ethics Statement

The University of Groningen ethics committee approved all the animal experiments described in this paper (permit numbers 5964A and 5964E) and the animals' discomfort was kept to a minimum.

### Mice and Bone Marrow Transplantation

Male mice deficient for *Cmklr1* on a C57BL/6J background and wild type (WT) littermates were kindly provided by MSD (Oss, the Netherlands) and kept on a 12-hour light/12-hour dark cycle, with *ad libitum* access to food and water. Starting at 3–4 months of age, they were fed a high fat, high cholesterol (HFC) diet, containing 21% fat from milk butter and 0.2% cholesterol (Scientific Animal Food and Engineering, Villemoisson-sur-Orge, France), for 12 weeks to induce NAFLD. To investigate the loss of *Cmklr1* in bone marrow-derived cells, female low-density lipoprotein receptor knock-out (*Ldlr^-/-^*) mice on a C57BL/6J background (bred in-house) were irradiated with 9.5 Gy [17] using X-Rad 320 (Precision X-ray, CT, USA) at 2–5 months of age. The next day, these mice were transplanted with bone marrow from WT or *Cmklr1^-/-^* mice by intravenous injection. To allow hematopoietic cells to replenish, the mice were allowed to recover on a regular chow diet after the transplantation procedure. After 10 weeks recovery, chimerism was confirmed with DNA isolated from whole blood (data not shown) and the *Ldlr^-/-^* mice were then fed a HFC diet for 12 weeks to induce NASH.

### Oral Glucose Tolerance Test and Insulin Tolerance Test

After 6 hours of fasting, the mice were subjected to an oral glucose tolerance test (OGTT) or an insulin tolerance test (ITT). For the OGTT, mice received a bolus of glucose (2 g/kg) and glucose was measured with a glucose meter in blood samples taken beforehand and at 15, 30, 60 and 120 minutes after gavage. For the ITT, mice were injected intraperitoneally with 0.7 U/kg insulin (WT and *Cmklr1^-/-^* mice) or 0.5 U/kg insulin (bone marrow-transplanted *Ldlr^-/-^* mice) (Actrapid, Novo Nordisk Canada Inc., Ontario, Canada) and blood glucose levels were measured at the same time points.

### Blood and Tissue Collection

Mice were euthanized by a heart puncture under general anesthesia for the collection of blood and tissues. Blood was spun at 3000 g for 10 minutes at 4°C and plasma was stored at −20°C. Tissues were snap-frozen in liquid nitrogen or fixed in 4% paraformaldehyde.

### Hepatic Lipid Extraction and Analysis

Lipids were extracted from crushed liver samples according to Bligh and Dyer's method [18]. Hepatic cholesterol and triglyceride levels were determined using commercially available kits (Roche, Mannheim, Germany).

### Plasma Measurements

Plasma cholesterol, triglycerides and free fatty acid levels were measured with commercially available kits (cholesterol and triglycerides: Roche; free fatty acids: DiaSys, Holzheim, Germany). Chemerin and insulin were determined in plasma from mice that had fasted for 6 hours, using commercially available ELISA kits (chemerin: R&D systems, Abingdon, UK; insulin: Alpco Diagnostics, Salem, NH).

### Real-time PCR

RNA from homogenized liver, visceral and subcutaneous adipose tissue (VAT and SAT) samples was isolated according to the manufacturer's instructions using Qiazol reagent (Qiagen, Venlo, the Netherlands). For real-time (RT)-PCR, cDNA was synthesized with a commercially available kit (Quantitect Reverse Transcription, Qiagen). RT-PCR was performed using Sybr Green Supermix (Bio-Rad, Veenendaal, the Netherlands) according to the manufacturer's instructions. The primer sequences are listed in Table S1.

### Histological Analysis

For histological analysis, paraffin-embedded liver, VAT and SAT sections (4 µm) were stained with Hematoxylin-Eosin (HE). HE-stained liver sections were scored for steatosis, lobular inflammation and hepatocyte ballooning by a board certified veterinary pathologist based on the Kleiner Scoring System [19]. The sum of these findings was used to determine the NAFLD activity score (NAS). HE-stained VAT and SAT sections were scanned using the NanoZoomer 2.0-HT slide scanner (Hamamatsu, Herrsching am Ammersee, Germany). To estimate adipocyte size, the number of adipocytes per mm^2^ were determined on scanned sections by manually counting all the adipocytes in an area of 4–5 mm^2^ using Aperio ImageScope (Leica Biosystems Imaging Inc., CA, USA). Frozen-cut liver sections (5 µm) were fixed in 4% paraformaldehyde and stained with antibodies against the macrophage markers CD68 and CD11b.

### Statistical Analysis

For statistical analysis, non-parametric t-tests were performed using GraphPad Prism 5.0 (San Diego, USA). Data were expressed as mean ± SEM and the threshold for significance was set at p<0.05.

## Results

### *Cmklr1* Deficiency Does not Affect Body Weight or Food Intake

**Figure 1:**
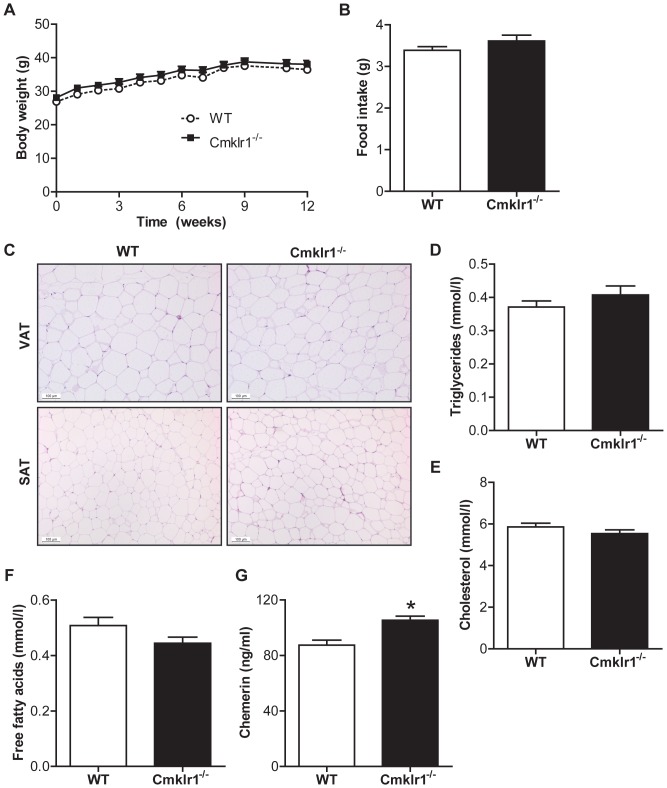
*Cmklr1* deficiency does not affect body weight, food intake or plasma lipid levels. Body weight (A) and food intake (B) were measured throughout the 12-week high fat, high cholesterol diet period. Food intake was expressed as average food intake per day. (C) Representative pictures were taken of Hematoxylin-Eosin (HE) stained visceral and subcutaneous adipose tissue (VAT and SAT) sections. Blood obtained by a heart puncture at the time of euthanasia was used to determine plasma triglycerides (D), plasma cholesterol (E) and plasma free fatty acids (F). (G) Chemerin levels were determined in blood obtained from the tail vein of mice that had fasted for 6 hours at the end of the diet period. Abbreviations: WT, wild type; *Cmklr1^-/-^*, chemokine-like receptor 1 knock-out. N = 7–8 for all experiments. Data are expressed as mean ± SEM. * p<0.05 vs WT.

As *Cmklr1* is known to be necessary for adipocyte differentiation [20] and *Cmklr1^-/-^* mice were shown to have reduced body weight and fat mass compared to WT mice [15], we evaluated body weight gain and food intake in mice fed a HFC diet for 12 weeks. No differences in body weight or food intake were observed between WT and *Cmklr1^-/-^* mice (Figs. 1A and 1B, respectively). Moreover, we observed no differences in the number of adipocytes per mm^2^ in visceral and subcutaneous adipose tissue (VAT and SAT) (VAT: WT, 143 cells/mm^2^±10; *Cmklr1^-/-^*, 155 cells/mm^2^±4, SAT: WT, 301 cells/mm^2^±10; *Cmklr1^-/-^*, 345 cells/mm^2^±31), indicating that adipose tissue morphology was similar in WT and *Cmklr1^-/-^* mice (Fig. 1C). In addition, plasma triglycerides (Fig. 1D), cholesterol (Fig. 1E) and free fatty acid levels (Fig. 1F) were the same in both genotypes. However, plasma chemerin levels were significantly increased in *Cmklr1^-/-^* mice fed a HFC diet compared to WT mice (Fig. 1G), suggesting a compensatory upregulation of the receptor ligand. To identify the origin of these increased plasma chemerin levels, we measured *chemerin* expression in liver, visceral and subcutaneous adipose tissue (VAT and SAT). *Chemerin* expression was significantly higher in VAT and SAT, but not in the liver (Fig. S1A). As chemerin has two other receptors, G protein coupled receptor 1 (*Gpr1*) and (C-C motif) receptor-like 2 (*Ccrl2*) [21], [22] we also measured whether the expression of these receptors was altered. No differences were found in the expression of these receptors in either liver tissue, VAT or SAT (Figs. S1B and S1C).

### *Cmklr1* Deficiency Does not Affect the Development of Systemic Insulin Resistance

**Figure 2:**
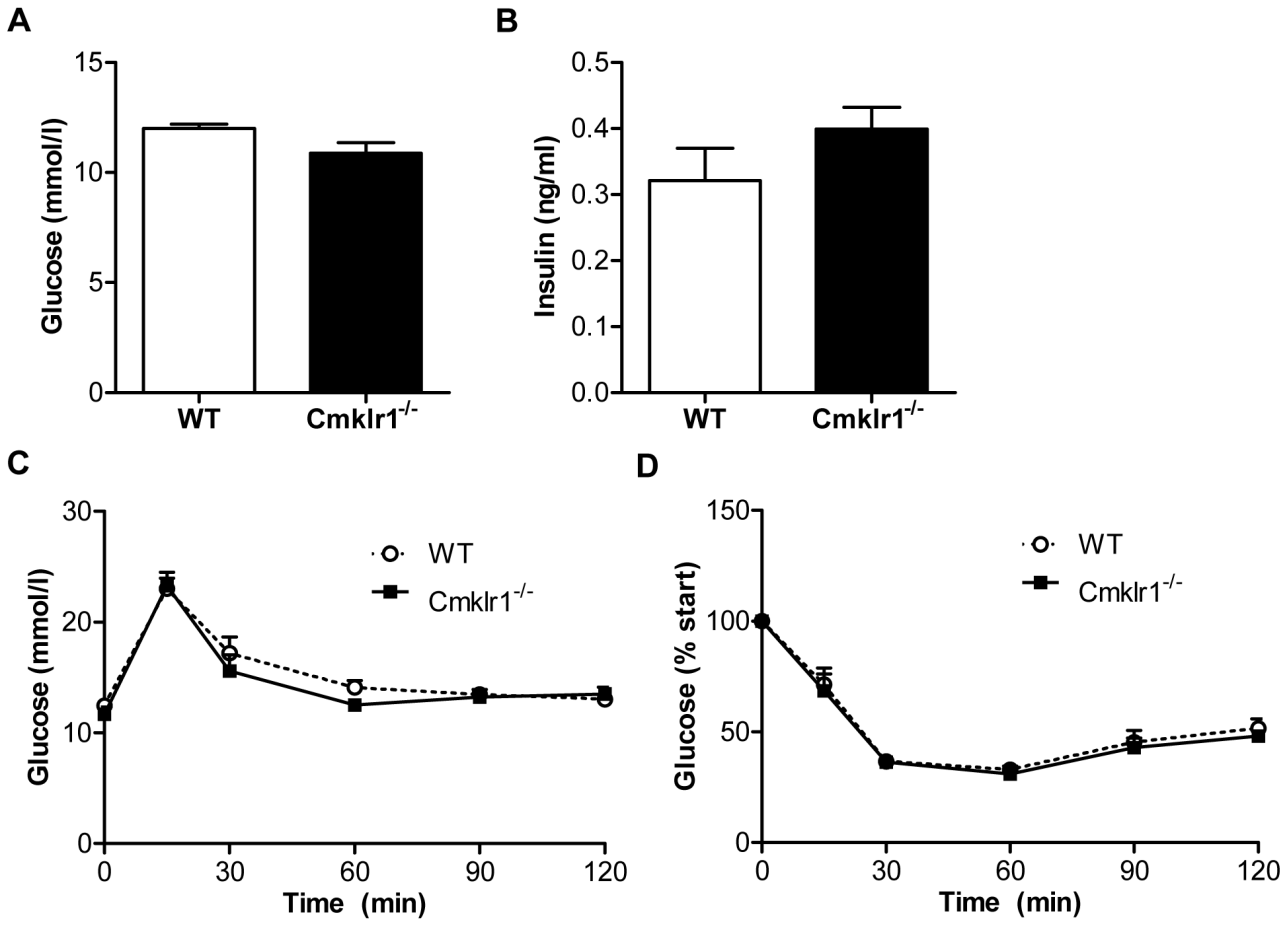
*Cmklr1* deficiency does not affect insulin resistance. To determine insulin resistance, glucose (A) and insulin levels (B) were measured in mice that had fasted for 6 hours at the end of the 12-week high fat, high cholesterol diet period. In addition, an oral glucose tolerance test (C) and an insulin tolerance test (D) were performed. Abbreviations: WT, wild type; *Cmklr1^-/-^*, chemokine-like receptor 1 knock-out. N = 7–8 for all experiments. Data are expressed as mean ± SEM. * p<0.05 vs WT.

As the chemerin-*Cmklr1* system has previously been implicated in the development of insulin resistance [15], [23], [24], we next assessed the glucose tolerance and insulin sensitivity in WT and *Cmklr1^-/-^* mice fed a HFC diet for 12 weeks. However, plasma glucose (Fig. 2A) and insulin levels (Fig. 2B) did not differ between the genotypes, and the mice responded similarly to an oral bolus of glucose (Fig. 2C) and an intraperitoneal insulin injection (Fig. 2D). This indicates that *Cmklr1* deficiency does not affect the glucose tolerance and insulin sensitivity of mice fed a HFC diet.

### *Cmklr1* Deficiency Does not Affect the Development of NAFLD

**Figure 3:**
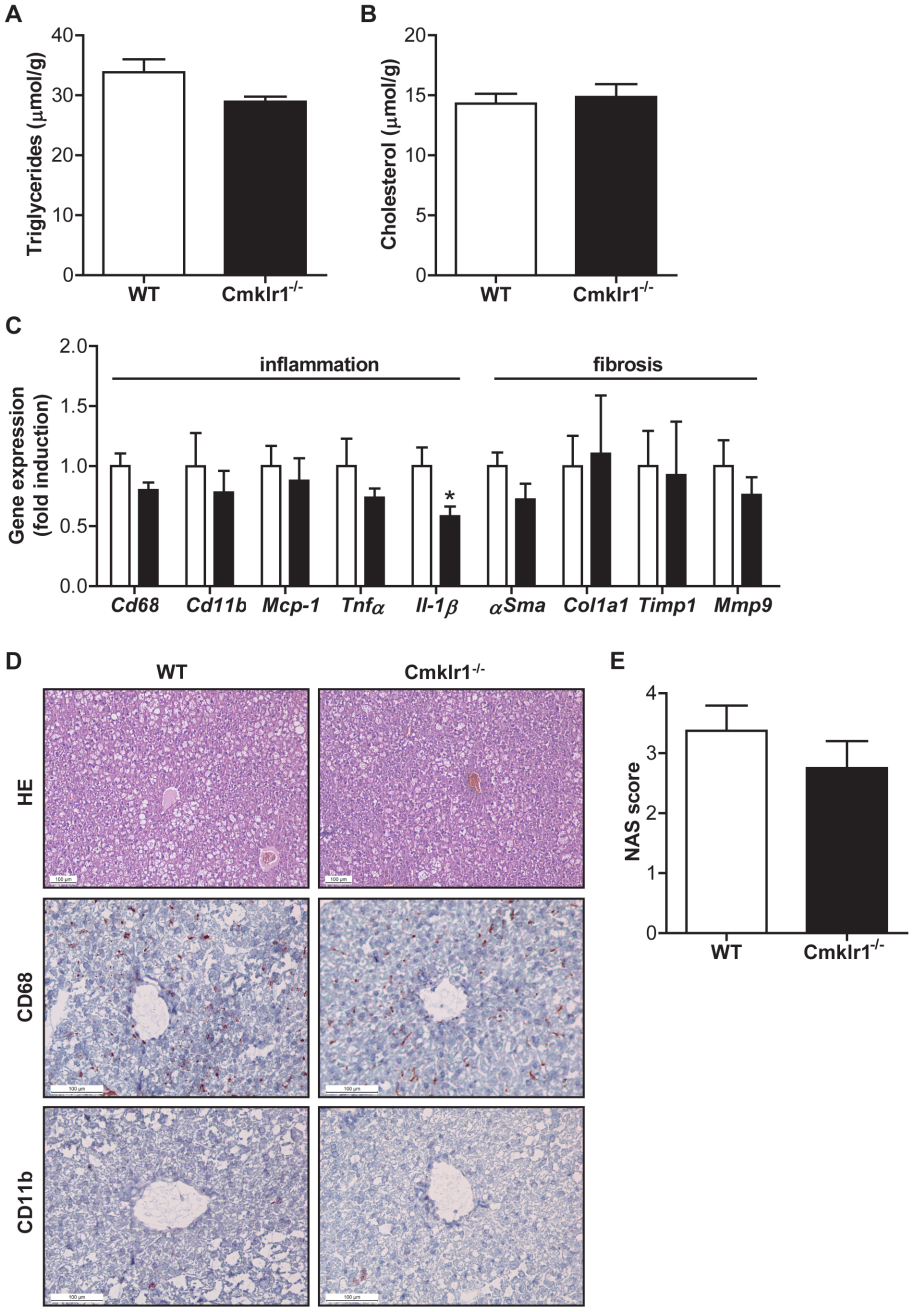
NAFLD progression is not affected by ablation of *Cmklr1*. To investigate the progression of NAFLD, hepatic triglyceride (A) and cholesterol (B) accumulation were measured in mice fed a high fat, high cholesterol diet for 12 weeks. (C) To determine the amount of inflammation and fibrosis in the liver, the inflammatory and pro-fibrotic gene expression were measured in WT mice (white bars) and *Cmklr1^-/-^* mice (black bars). (D) In addition, a staining for the macrophage markers CD68 and CD11b was performed on frozen-cut liver sections. (E) NAFLD Activity Score (NAS) was determined by pathological examination of Hematoxylin-Eosin (HE) stained liver sections. Abbreviations: WT, wild type; *Cmklr1^-/-^*, chemokine-like receptor 1 knock-out; *Cd68*, cluster of differentiation 68; *Cd11b*, alpha M integrin (*Mac1*); *Mcp-1*, monocyte chemo-attractant protein 1; *Tnfα*, tumor necrosis factor α; *Il1-β*, interleukin 1β; *αSma*, α-smooth muscle actin; *Col1a1*, collagen type 1 alpha 1; *Timp1*, tissue inhibitor of metalloproteinase 1; *Mmp9*, matrix metallopeptidase 9. N = 7–8 for all experiments. Data are expressed as mean ± SEM. * p<0.05 vs WT.

To investigate whether *Cmklr1* plays a role in the development of diet-induced NAFLD, we analyzed lipid levels and gene expression in the livers of *Cmklr1^-/-^* mice fed a HFC diet for 12 weeks. Although liver weight, expressed as a percentage of body weight, was lower in *Cmklr1^-/-^* mice (WT, 5.70%±0.21; *Cmklr1^-/-^*, 5.16%±0.041, p<0.05), hepatic triglyceride content did not differ between WT and *Cmklr1^-/-^* mice (Fig. 3A). In line with this, no difference in hepatic cholesterol was observed between the genotypes (Fig. 3B). The expression of genes encoding for proteins involved in macrophage activation and infiltration (*Cd68* and *Cd11b*), inflammation (*Mcp-1* and *Tnfα*) and fibrosis (*αSma*, *Col1a1*, *Timp1* and *Mmp9*) was similar in *Cmklr1^-/-^* mice and WT mice (Fig. 3C). However, *Il-1β* gene expression was slightly, but significantly, reduced in *Cmklr1^-/-^* mice (Fig. 3C). Furthermore, immunostaining for CD68 and CD11b demonstrated that *Cmklr1* deficiency did not affect the activation or infiltration of macrophages in the livers of mice fed a HFC diet (Fig. 3D). Pathological examination of HE-stained liver sections (Fig. 3D) confirmed these findings (Figs. S2A-C) and revealed no difference in NAS (Fig. 3E).

### Hematopoietic Deletion of *Cmklr1* Does not Affect the Development of Insulin Resistance or NASH

**Figure 4:**
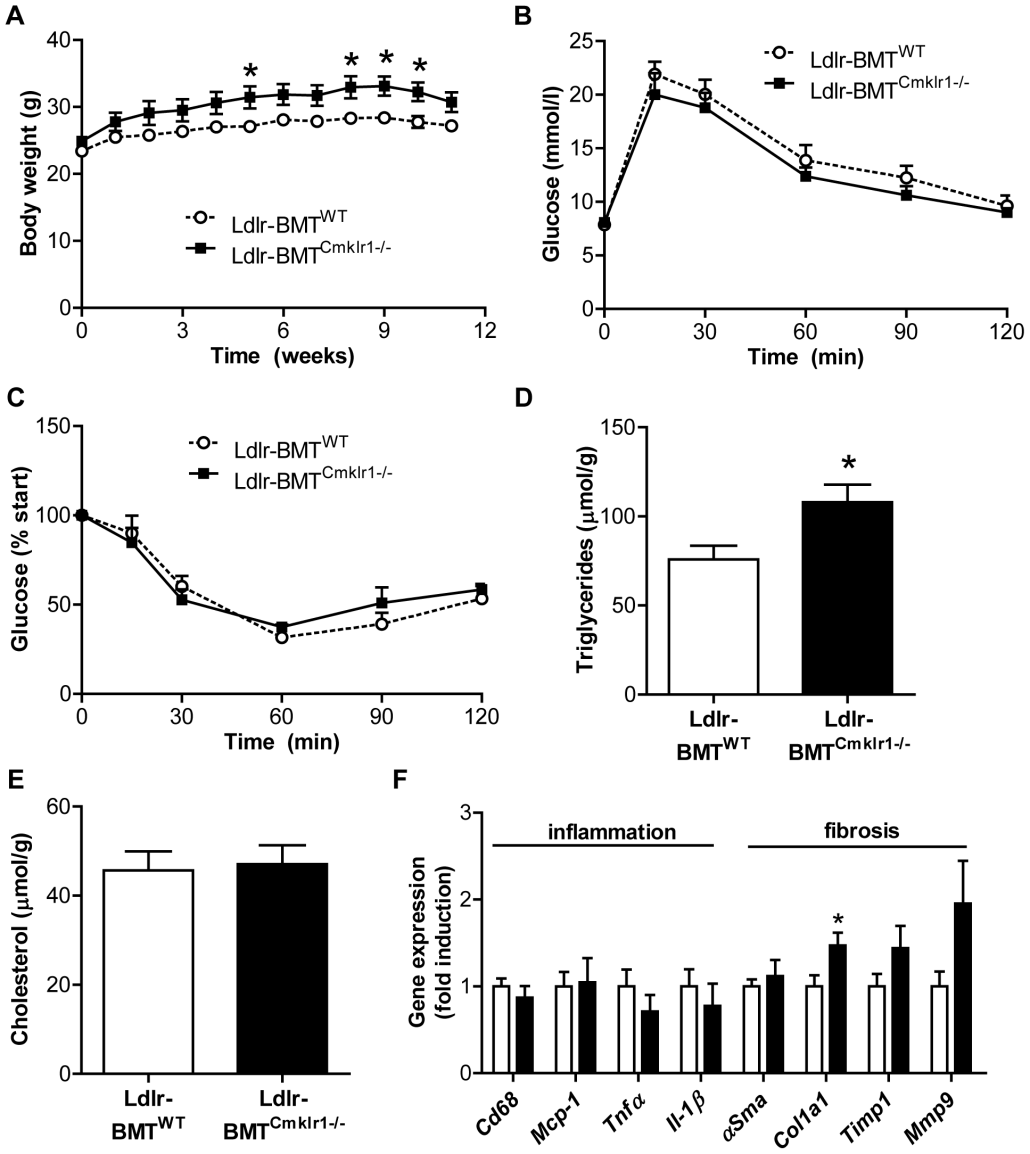
Hematopoietic deletion of *Cmklr1* does not affect NASH development in *Ldlr^-/-^* mice. (A) Body weight of *Ldlr*-BMT*^WT^* and *Ldlr*-BMT*^Cmklr1-/-^* mice was measured during the high fat, high cholesterol diet period of 12 weeks. Insulin resistance was assessed by an oral glucose tolerance test (B) and an insulin tolerance test (C). NASH progression was investigated by measuring hepatic triglyceride (D) and cholesterol (E) accumulation and by determining hepatic inflammatory and pro-fibrotic gene expression (F) in *Ldlr*-BMT^WT^ mice (white bars) and *Ldlr*-BMT*^Cmklr1-/-^* mice (black bars). Abbreviations: *Ldlr*-BMT*^WT^*, low-density lipoprotein receptor knock-out mice transplanted with wild type bone marrow cells; *Ldlr*-BMT*^Cmklr1-/-^*, low-density lipoprotein receptor knock-out mice transplanted with chemokine-like receptor 1 knock-out bone marrow cells; *Cd68*, cluster of differentiation 68; *Mcp-1*, monocyte chemo-attractant protein 1; *Tnfα*, tumor necrosis factor α; *Il1-β*, interleukin 1β; *αSma*, α-smooth muscle actin; *Col1a1*, collagen type 1 alpha 1; *Timp1*, tissue inhibitor of metalloproteinase 1; *Mmp9*, matrix metallopeptidase 9. N = 5–7 for all experiments. Data are expressed as mean ± SEM. * p<0.05 vs WT.

To further investigate if *Cmklr1* deficiency affects the progression of insulin resistance and NAFLD, we transplanted bone marrow from *Cmklr1^-/-^* mice or WT mice into *Ldlr^-/-^* mice. *Ldlr^-/-^* mice fed a HFC diet can be regarded as a model for NASH that is specifically driven by Kupffer cell activation and the recruitment of macrophages [25], [26]. *Ldlr^-/-^* mice transplanted with *Cmklr1^-/-^* bone marrow cells (*Ldlr*-BMT*^Cmklr1-/-^*) had a greater gain in body weight than *Ldlr^-/-^* mice transplanted with WT bone marrow cells (*Ldlr*-BMT^WT^) (Fig. 4A), and this could not be explained by differences in food intake (Fig. S3A). In concordance, the number of adipocytes per mm^2^ was lower in *Ldlr*-BMT*^Cmklr1-/-^* mice compared to *Ldlr*-BMT^WT^ mice in both VAT (*Ldlr*-BMT^WT^, 245 cells/mm^2^±26; *Ldlr*-BMT*^Cmklr1-/-^*, 178 cells/mm^2^±15; p = 0.07) and SAT (*Ldlr*-BMT^WT^, 474 cells/mm^2^±51; *Ldlr*-BMT*^Cmklr1-/-^*, 302 cells/mm^2^±30; p<0.05), indicating that *Ldlr*-BMT*^Cmklr1-/-^* mice have larger adipocytes (Fig. S3B). In contrast, we found no significant differences in plasma triglyceride, cholesterol, glucose and insulin levels between the two groups of mice (Figs. S3C-F). Moreover, hematopoietic deletion of *Cmklr1* did not affect glucose tolerance (Fig. 4B) or insulin tolerance (Fig. 4C). Whereas liver weight, expressed as a percentage of body weight, was slightly lower in *Ldlr*-BMT*^Cmklr1-/-^* mice compared to *Ldlr*-BMT^WT^ mice (*Ldlr*-BMT^WT^, 5.49%±0.086; *Ldlr*-BMT*^Cmklr1-/-^*, 5.08%±0.10, p<0.05), the *Ldlr*-BMT*^Cmklr1-/-^* mice showed a small increase in their hepatic triglyceride content and hepatic steatosis (Fig. 4D and Fig. S3G). No differences were observed in hepatic cholesterol accumulation (Fig. 4E). As in the whole-body knock-out model, no differences in inflammatory gene expression (*Cd68*, *Mcp-1*, *Tnfα* and *Il-1β*) were observed (Fig. 4F). Examination of pro-fibrotic genes revealed a trend towards an increase of fibrosis in *Ldlr-*BMT*^Cmklr1-/-^* mice. However, the differences were only significant for *Col1a1* (Fig. 4F). Overall, these data indicate that whole-body or hematopoietic ablation of *Cmklr1* does not impact on the development of systemic insulin resistance and NAFLD in mice.

## Discussion

The role of *Cmklr1* in the development of insulin resistance and NAFLD is controversial. Our data show that whole-body as well as hematopoietic deletion of *Cmklr1* in *Ldlr^-/-^* mice did not affect the development of insulin resistance or NAFLD when the mice were fed a HFC-diet for 12 weeks. These results raise the question whether the alterations in serum chemerin levels and hepatic chemerin and *Cmklr1* expression found in rodent models of NAFLD [7], [12], [14] are causally involved in the development and/or progression of this disease.

In contrast to our findings, others previously reported that *Cmklr1^-/-^* mice have reduced hepatic inflammation compared to WT mice [15], which may be related to the reduced body weight and fat mass found in these *Cmklr1* ^-/-^ mice. In line with this, the same investigators showed that *Cmklr1^-/-^* mice have reduced steatosis on a low fat diet. However, these differences were not present on a high fat diet [15]. Our results confirm their latter findings. Consistent with our results, another paper observed no differences in body weight, fat mass and glucose tolerance between WT and *Cmklr1^-/-^* mice at a young age. In that study, *Cmklr1^-/-^* mice started to gain more fat mass than WT mice only from age 8 months onwards [16]. The increased body weight gain (Fig. 4A) and adipocyte size (Fig. S3B) found in *Ldlr^-/-^* mice with a hematopoietic deletion of *Cmklr1* might therefore be an age-induced effect. These mice were euthanized at 7–10 months of age, whereas the whole-body knock-outs were euthanized at 6–7 months of age. These results suggest that hematopoietic cells might be responsible for the age-induced weight gain of *Cmklr1* ^-/-^ mice. In contrast to *Cmklr1^-/-^* mice, the *Ldlr*-BMT*^Cmklr1-/-^* mice had increased hepatic triglyceride levels (Fig. 4D), increased hepatic steatosis (Fig. S3G) and a tendency towards an increased expression of pro-fibrotic genes (Fig 4F). However, these differences were small and may well be explained by the increased body weight gain seen in these mice.

As neither whole-body *Cmklr1* deficiency and hematopoietic deletion of *Cmklr1* in *Ldlr^-/-^* mice affected the development of NAFLD, the alterations in chemerin and *Cmklr1* levels found in humans and mouse models of NAFLD may be a secondary effect of the metabolic syndrome and NAFLD [10]–[12], [14]. In these disorders, many of the factors that can regulate chemerin and *Cmklr1* expression become dysregulated. First, chemerin is an adipokine and its secretion rises with increasing adiposity. Second, plasma TNFα levels and *Tnf*α expression in adipose tissue and the liver are higher in NASH [27], [28]. This cytokine has been shown to increase chemerin expression from adipose tissue [29], [30] and to modulate chemerin activity [31]. Finally, FXR, a nuclear receptor that regulates glucose and lipid homeostasis, induces chemerin expression [12]. The alterations in chemerin levels found in rodent models of NAFLD may thus be caused by increased adiposity, increased *Tnf*α levels [30] or altered FXR activity in NAFLD. Expression of *Cmklr1* in hepatocytes is upregulated by adiponectin [14]. This adipokine protects against steatosis and inflammation and is reduced in human patients with NAFLD [32]. Thus, the reduced expression of *Cmklr1* in patients with NAFLD may be explained by reduced adiponectin levels and it may not be causally involved in the development of NAFLD.

A few limitations to our study must be taken into account. In our model we studied the effects of the absence of *Cmklr1*, but the other receptors that can bind chemerin, *Gpr1* and *Ccrl2*, were still present [21], [22]. It is possible that the increased plasma chemerin levels found in our *Cmklr1^-/-^* mice (Fig. 1G), which most likely originate from the adipose tissue and not from the liver (Fig. S1A), act on these receptors to compensate for the loss-of-function of *Cmklr1*. So far, however, no signaling function has been described for these receptors [33]. Moreover, we did not find any differences in the expression of *Gpr1* and *Ccrl2* in liver, VAT or SAT between WT and *Cmklr1^-/-^* mice, indicating that there is no compensatory upregulation of these receptors due to *Cmklr1* deficiency (Figs. S1B and S1C). In addition to the other chemerin receptors, *Cmklr1* has another ligand, named resolvin E1 (RvE1). RvE1 is derived from omega-3 polyunsaturated fatty acids and has been described to inhibit NF-κB activity via *Cmklr1* [34]. RvE1 has also been shown to reduce fat accumulation and macrophage infiltration in the liver and to protect against hepatocyte death [35]. This makes it even more remarkable that *Cmklr1* deficiency does not appear to affect NAFLD development. To fully elucidate the role of *Cmklr1* in NAFLD, experiments in mice deficient for chemerin, RvE1, or both, need to be performed.

In summary, our results show that whole-body and hematopoietic deletion of *Cmklr1* in *Ldlr^-/-^* mice do not affect the development of systemic insulin resistance and NAFLD in mice. This makes it less likely that the alterations in chemerin and *Cmklr1* levels found in mouse models of NAFLD [12], [14] are causally related to the development and/or progression of this disease. We feel the associations between chemerin or *Cmklr1* levels and NAFLD should therefore be interpreted with caution.

## Supporting Information

Figure S1 **The expression of chemerin is increased in adipose tissue, but not in the liver.** The expression of *chemerin* (A) and its receptors, *Gpr1* (B) and *Ccrl2* (C), was measured in liver, visceral and subcutaneous adipose tissue of WT mice (white bars) and *Cmklr1^-/-^* mice (black bars) fed a high fat, high cholesterol diet for 12 weeks. Abbreviations: WT, wild type; *Cmklr1^-/-^*, chemokine-like receptor 1 knock-out; VAT, visceral adipose tissue; SAT, subcutaneous adipose tissue; *Gpr1*, G protein-coupled receptor 1; *Ccrl2*, (C-C) motif receptor-like 2. N = 6-8 for all experiments. Data are expressed as mean ± SEM.
(TIF)

Figure S2 **Steatosis, lobular inflammation and ballooning scores were not affected by** ***Cmklr1*** **deficiency.** Hematoxylin-Eosin (HE) stained liver sections of mice fed a high fat, high cholesterol diet for 12 weeks were scored for steatosis (A), lobular inflammation (B) and ballooning (C) by a certified veterinary pathologist. Abbreviations: WT, wild type; *Cmklr1^-/-^*, chemokine-like receptor 1 knock-out. N = 8 for all experiments. Data are expressed as mean ± SEM.
(TIF)

Figure S3 **Characteristics of bone marrow transplanted** ***Ldlr^-/-^*** **mice.** (A) Food intake was measured throughout the 12-week high fat, high cholesterol (HFC) diet period and calculated in grams per day. (B) Representative pictures were taken of Hematoxylin-Eosin (HE) stained visceral and subcutaneous adipose tissue (VAT and SAT) sections. Plasma triglycerides (C), cholesterol (D), glucose (E) and insulin (F) levels were determined after 12 weeks of HFC feeding. (G) Paraffin-embedded liver sections were stained with HE for histological analysis. Abbreviations: *Ldlr*-BMT*^WT^*, low-density lipoprotein receptor knock-out mice transplanted with wild type bone marrow cells; *Ldlr*-BMT*^Cmklr1-/-^*, low-density lipoprotein receptor knock-out mice transplanted with chemokine-like receptor 1 knock-out bone marrow cells. N = 5–7 for all experiments. Data are expressed as mean ± SEM.
(TIF)

Table S1 **Primer sequences.**
(DOCX)
