# Supplementary figures and images for: Chemokine-Like Receptor 1 Deficiency Does Not Affect the Development of Insulin Resistance and Nonalcoholic Fatty Liver Disease in Mice
Source: PLoS One. 2014 Apr 29;9(4):e96345. doi: 10.1371/journal.pone.0096345 (PMC4004559; doi:10.1371/journal.pone.0096345)

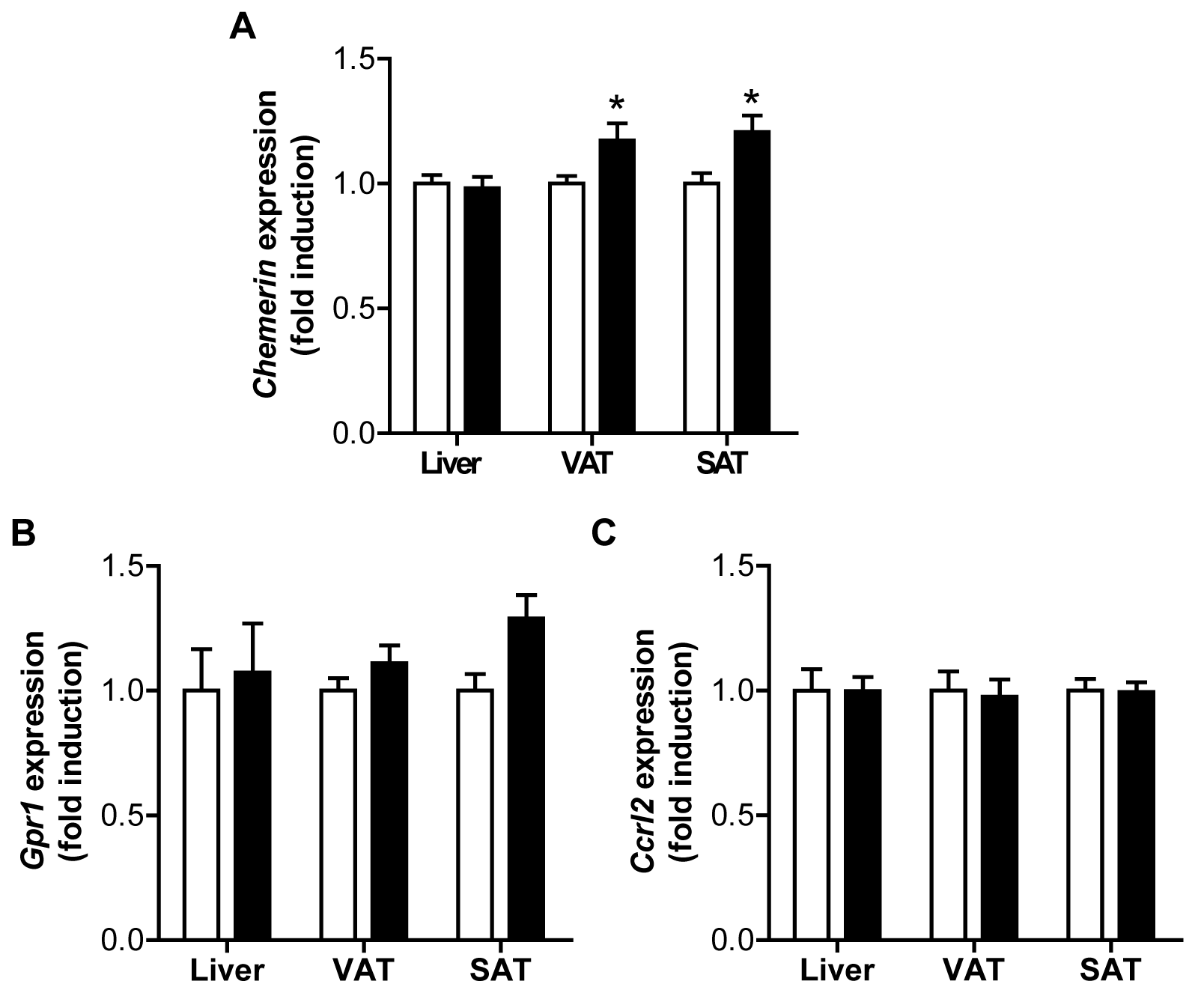

Supplement: Figure S1 — The expression of chemerin is increased in adipose tissue, but not in the liver. The expression of chemerin (A) and its receptors, Gpr1 (B) and Ccrl2 (C), was measured in liver, visceral and subcutaneous adipose tissue of WT mice (white bars) and Cmklr1-/- mice (black bars) fed a high fat, high cholesterol diet for 12 weeks. Abbreviations: WT, wild type; Cmklr1-/-, chemokine-like receptor 1 knock-out; VAT, visceral adipose tissue; SAT, subcutaneous adipose tissue; Gpr1, G protein-coupled receptor 1; Ccrl2, (C-C) motif receptor-like 2. N = 6-8 for all experiments. Data are expressed as mean ± SEM. (TIF) [file pone.0096345.s001.tif]

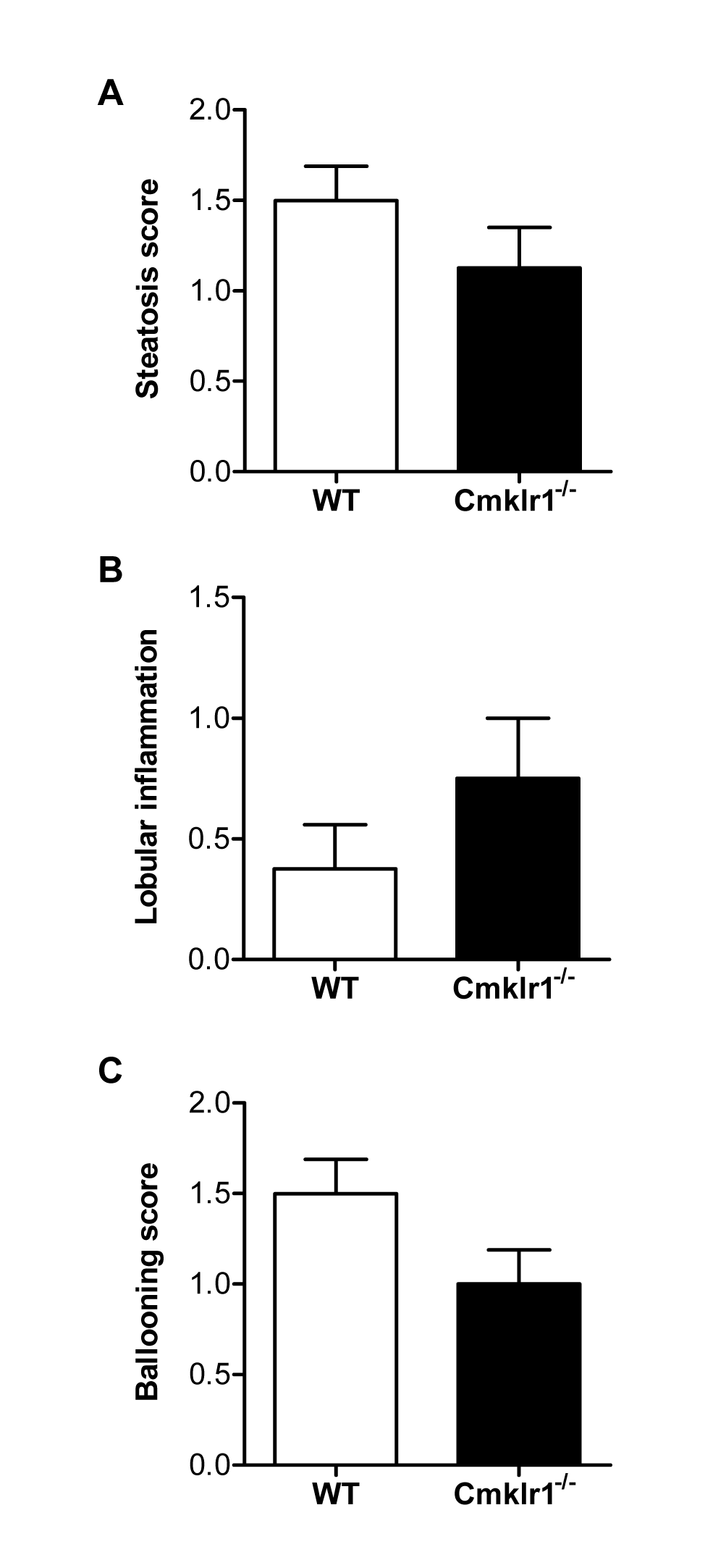

Supplement: Figure S2 — Steatosis, lobular inflammation and ballooning scores were not affected by Cmklr1 deficiency. Hematoxylin-Eosin (HE) stained liver sections of mice fed a high fat, high cholesterol diet for 12 weeks were scored for steatosis (A), lobular inflammation (B) and ballooning (C) by a certified veterinary pathologist. Abbreviations: WT, wild type; Cmklr1-/-, chemokine-like receptor 1 knock-out. N = 8 for all experiments. Data are expressed as mean ± SEM. (TIF) [file pone.0096345.s002.tif]

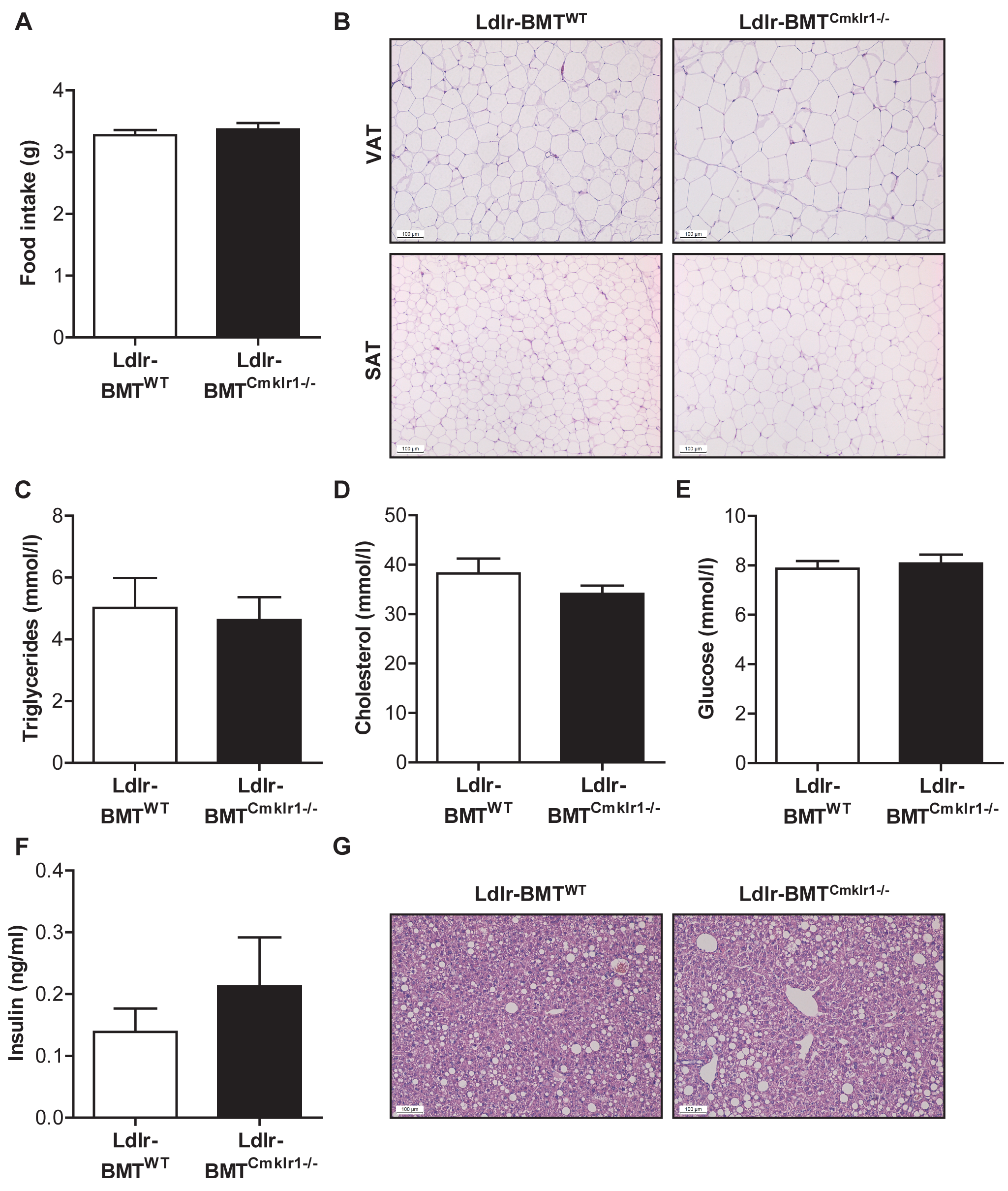

Supplement: Figure S3 — Characteristics of bone marrow transplanted Ldlr-/- mice. (A) Food intake was measured throughout the 12-week high fat, high cholesterol (HFC) diet period and calculated in grams per day. (B) Representative pictures were taken of Hematoxylin-Eosin (HE) stained visceral and subcutaneous adipose tissue (VAT and SAT) sections. Plasma triglycerides (C), cholesterol (D), glucose (E) and insulin (F) levels were determined after 12 weeks of HFC feeding. (G) Paraffin-embedded liver sections were stained with HE for histological analysis. Abbreviations: Ldlr-BMTWT, low-density lipoprotein receptor knock-out mice transplanted with wild type bone marrow cells; Ldlr-BMTCmklr1-/-, low-density lipoprotein receptor knock-out mice transplanted with chemokine-like receptor 1 knock-out bone marrow cells. N = 5–7 for all experiments. Data are expressed as mean ± SEM. (TIF) [file pone.0096345.s003.tif]
