# Supplementary material for: Chemokine-Like Receptor 1 Deficiency Does Not Affect the Development of Insulin Resistance and Nonalcoholic Fatty Liver Disease in Mice
Source: PLoS One. 2014 Apr 29;9(4):e96345. doi: 10.1371/journal.pone.0096345 (PMC4004559; doi:10.1371/journal.pone.0096345)
Supplement: Table S1 — Primer sequences. (DOCX) [file pone.0096345.s004.docx]

**Supporting information**

**Table S1. Primer sequences.**

| Gene | Forward primer 5’-3’ | Reverse primer 5’-3’ |
| --- | --- | --- |
| *Chemerin* | CCAACTGCCCCAAGAAGGA | CGCCTTCTCCCGTTTGGT |
| *Gpr1* | GGAGCTCAGCATTCATCACA | GACAGGCTCTTGGTTTCAGC |
| *Ccrl2* | TTCCAACATCCTCCTCCTTG | GATGCACGCAACAATACCAC |
| *Cd68* | TGACCTGCTCTCTCTAAGGCTACA | TCACGGTTGCAAGAGAAACATG |
| *Cd11b* | TCAGAGAATGTCCTCAGCAG | TGAGACAAACTCCTTCATCTTC |
| *Mcp-1* | GCTGGAGAGCTACAAGAGGATCA | ACAGACCTCTCTCTTGAGCTTGGT |
| *Tnfα* | CATCTTCTCAAAATTCGAGTGACAA | TGGGAGTAGACAAGGTACAACCC |
| *Il-1β* | TGCAGCTGGAGAGTGTGG | TGCTTGTGAGGTGCTGATG |
| *αSma* | ACGAACGCTTCCGCTGC | GATGCCCGCTGACTCCAT |
| *Col1a1* | AACCCTGCCCGCACATG | CAGACGGCTGAGTAGGGAACA |
| *Timp1* | CGCCTAAGGAACGGAAATTTG | AGGGATAGATAAACAGGGAAACACTGT |
| *Mmp9* | CCTGGAACTCACACGACATCTTC | TGGAAACTCACACGCCAGAA |
